# Experimental hierarchy of two-qubit quantum correlations without state tomography

**DOI:** 10.1038/s41598-023-35015-9

**Published:** 2023-05-26

**Authors:** Shilan Abo, Jan Soubusta, Kateřina Jiráková, Karol Bartkiewicz, Antonín Černoch, Karel Lemr, Adam Miranowicz

**Affiliations:** 1 Institute of Spintronics and Quantum Information, Faculty of Physics, Adam Mickiewicz University, 61-614 Poznań, Poland; 2 Palacký University Olomouc, Faculty of Science, Joint Laboratory of Optics of PU and IP CAS, 17. listopadu 1192/12, 779 00 Olomouc, Czech Republic; 3 Institute of Physics of the Czech Academy of Sciences, Joint Laboratory of Optics of PU and IP CAS, 17. listopadu 1154/50a, 779 00 Olomouc, Czech Republic

**Keywords:** Optical physics, Quantum physics

## Abstract

A Werner state, which is the singlet Bell state affected by white noise, is a prototype example of states, which can reveal a hierarchy of quantum entanglement, steering, and Bell nonlocality by controlling the amount of noise. However, experimental demonstrations of this hierarchy in a sufficient and necessary way (i.e., by applying measures or universal witnesses of these quantum correlations) have been mainly based on full quantum state tomography, corresponding to measuring at least 15 real parameters of two-qubit states. Here we report an experimental demonstration of this hierarchy by measuring only six elements of a correlation matrix depending on linear combinations of two-qubit Stokes parameters. We show that our experimental setup can also reveal the hierarchy of these quantum correlations of generalized Werner states, which are any two-qubit pure states affected by white noise.

## Introduction

Quantum correlations reveal not only the strangeness of quantum mechanics, but are the main resources for quantum technologies, including quantum sensing and quantum information processing^1^. Thus, the detection, control, and quantification of these resources are of paramount importance.

Among different types of quantum correlations, a special interest has been paid to quantum entanglement^2^, Einstein–Podolsky–Rosen (EPR) steering (also called quantum steering)^3,4^, and Bell nonlocality that can be revealed by testing the violation of a Bell inequality^5^. These types of correlations coincide for two-qubit pure states, but can be different for mixed states. Probably, the most intuitive distinction between these three types of quantum correlations for two systems (parties) can be given from a cryptographic perspective with the use of trusted and untrusted detectors. Specifically, according to Refs.^6,7^: (i) quantum entanglement can be revealed if both parties use only trusted detectors, (ii) EPR steering can be tested if one party uses trusted detectors and the other untrusted ones, and (iii) Bell nonlocality can be demonstrated if both parties use untrusted detectors. We experimentally determined and compared measures of these correlations for Werner states.

It is theoretically well known that by gradually adding noise to a pure state, one can reveal a hierarchy of different types of quantum correlations, including quantum entanglement, EPR steering, and Bell nonlocality. These effects are equivalent for two-qubit pure states, however they are in general different for mixed states. Werner^8^ found in 1989 that the singlet Bell state affected by white noise can be entangled without exhibiting Bell nonlocality, i.e., without violating any Bell inequality. It was further found that Werner states with a proper amount of white noise can be entangled but unsteerable, or steerable without exhibiting Bell nonlocality, in addition to the trivial cases when a given state is Bell nonlocal (so also steerable and entangled) or separable (so also unsteerable and Bell local). Even a more refined hierarchy can be revealed by considering generalized Werner states, defined as mixtures of an arbitrary two-qubit pure states and white noise^9^. Thus, the Werner and Werner-like states can be considered prototype examples of states indicating such a hierarchy. Their generation and the detection of their quantum correlations are a central topic of this paper.

Here we study a hierarchy of quantum correlations via their measures. We note that various other hierarchies of non-universal witnesses of quantum correlations have been investigated in detail. These include studies of sufficient conditions (i.e., nonuniversal witnesses) for observing specific types of correlations via matrices of moments of, e.g., the annihilation, creation, position, momentum, or Pauli operators. For example: (i) hierarchies of various witnesses of spatial^10^ and spatiotemporal^11,12^ correlations of bosonic systems revealing their nonclassicality via a nonpositive Glauber–Sudarshan *P* function; (ii) a hierarchy of entanglement witnesses^13,14^ based on the Peres–Horodecki partial transposition criterion or their generalizations^15^ using contraction maps (e.g., realignment) and positive maps (e.g., those of Kossakowski, Choi, and Breuer); (iii) a hierarchy of necessary conditions for the correlations that arise when performing local measurements on separate quantum systems, which enabled finding a hierarchy of upper bounds on Bell nonlocality^16,17^ (iv) a hierarchy of EPR steering witnesses^18^ based on entanglement criteria with the constraint that measurement devices of one party cannot be trusted. Especially powerful methods for finding infinite hierarchies of quantum-correlation criteria are those formulated as semidefinite programs^16–20^. Note that semidefinite programming has been found very effective in calculating not only nonuniversal witnesses but also measures (or universal witnesses) of quantum steering^3,21,22^, Bellnonlocality^5^, and entanglement^2^. It could also be noted that a hierarchy of quantum nonbreaking channels, which is closely related to a hierarchy of temporal^23^ and spatial quantum correlations, has been studied very recently both theoretically and experimentally in Ref.^24^, where the effects of white noise (or, equivalently, of a qubit-depolarizing channel) on quantum memory, temporal steerability^25,26^, and nonmacrorealism were revealed by applying a *full* quantum process tomography.

The use of measures or universal witnesses of these quantum correlations, however, is required to demonstrate experimentally such hierarchies in a sufficient and necessary manner. For example, to our knowledge, no experiment has been performed to determine standard entanglement measures of a general two-qubit mixed state without full quantum state tomography (QST). These measures include the concurrence^27^, which is a measure of the entanglement of formation, the negativity^28^ related to the Peres–Horodecki entanglement criterion, and the relative entropy of entanglement^29^. Thus, a QST-based approach to study a hierarchy of quantum correlations was applied in our former related study^9^, which was based on measuring 16 real parameters for two-qubit Werner states.

A hierarchy of quantum-correlation measures enables efficient estimations of one measure for a given value of another. More specifically, the estimations of a measure of a given type of quantum correlation for a certain value of a measure (or bounds) of another type of quantum correlations were reported for arbitrary or specific classes of two-qubit states. These estimations include various comparisons of: (i) entanglement and Bell nonlocality^30–33^, (ii) steering and Bell nonlocality^34^, as well as (iii) entanglement and steering^35^. Note that such estimations can also be applied to compare non-equivalent measures describing the same type of correlations, including two-qubit entanglement^36,37^ or single-qubit nonclassicality^38^. Explorations of the relationships between measures of entanglement, steering, and Bell nonlocality for specific types of two-qubit states have also been attracting a considerable interest. Recent studies include, e.g., theoretical analyses of two-qubit *X*-states^39^ and two-mode Gaussian states^40^. Experimental QST-based hierarchies of quantum entanglement, steering, and Bell nonlocality for specific classes of two-qubit states in relation to the above-mentioned estimations were also reported, which include experiments with mixtures of partially entangled two-qubit pure states^41^ and GWSs based on full QST^9^ or full quantum process tomography^24^. Such a hierarchy for the Werner states is also experimentally studied here but *without* applying a full QST.

We note that an experimental method for testing polarization entanglement without QST of general two qubit states was proposed in Ref.^42^ based on measuring a collective universal witness of Ref.^43^. However, the method, to our knowledge, has not been implemented experimentally yet. Another experimental approach to determine entanglement of a given state without QST can be based on measuring a bipartite Schmidt number, which satisfies various conditions of a good entanglement measure^44,45^ and can be determined experimentally via a witnessing approach^46^. However, it is not clear how the same method can also be used to experimentally determine steering and nonlocality measures. Note that we want to apply a versatile experimental setup, which can be used to determine various measures of all the three types of quantum correlations.

Multiple indicators of quantum steering have been demonstrated experimentally (for a review see Ref.^4^). We note a very recent Ref.^47^, where it was shown experimentally that a critical steering radius is the most powerful among practical steering indicators. Its scaling property allows classifying as steerable or non-steerable various families of quantum states. This approach is useful in testing theoretical concepts of the critical radius in real experiments prone to unavoidable noise. The authors used a setup introducing losses and measured elements of a correlation matrix to determine the steering indicators. Similar quantifiers, but describing nonlocality and entanglement, were measured in Ref.^48^ using the parameters *M* and *F*, which are also applied in this paper.

Here, we report the first (to our knowledge) experimental demonstration of the hierarchy of measures of entanglement, steering, and Bell nonlocality without applying full QST, i.e., by measuring only six elements of a correlation matrix *R* (corresponding to linear combinations of two-qubit Stokes parameters) for the Werner states. Moreover, we show that the generalized Werner states (GWSs), which are mixtures of an arbitrary two-qubit pure state and white noise, can reveal a more refined hierarchy of the quantum-correlation measures using our experimental setup.

We note that the setup applied in this work was also used earlier in Refs.^48–51^, but for conceptually different tasks, e.g., measuring collective nonlinear witnesses of entanglement^52,53^, Bell nonlocality measure^54^, or diagnosing an entanglement-swapping protocol. Moreover, the setup enables entanglement swapping and measuring multicopy entanglement witnesses as inspired by Refs.^55,56^.

The setup also bears some similarities with a previously proposed and implemented scheme by Bovino et al.^56^. Our experimental method of measuring *R* for general two qubit states is conceptually similar to that reported in Ref.^56^ for measuring a nonlinear entropic witness. We find that the witness, defined in the next section, can actually be interpreted as the three-measurement steering measure *S*. However, the advantage of our method is that it is more versatile. As shown in Ref.^48^, one can perform a full tomography of all the elements of the *R* matrix rather than only determining its trace. Thus, from the set of six numbers (determining a correlation matrix *R*) we can learn much more about quantum correlations compared to the original method of Ref.^56^. In addition to that, our design provides several practical benefits with respect to Ref.^56^. Namely from the experimental point of view, it only requires a single Hong–Ou–Mandel interferometer instead of two. Moreover, our design shares the same geometry with the entanglement-swapping protocol^48^. As a result, it can be deployed in future teleportation-based quantum networks to acquire various entanglement measures of distributed quantum states.

This experimental method of measuring the *R* matrix enables us a complete determination of not only steering measures, but also a fully entangled fraction (FEF)^57^ and Bell nonlocality measures^58^. We note that for the GWSs, the FEF is exactly equal to the two most popular measures of entanglement, i.e., the negativity and concurrence^2^. Thus, the hierarchy of the three measures can be experimentally determined from the *R* matrix for the Werner states, which is the main goal of this paper.

## Correlation matrix *R* for Werner and Werner-like states

We study quantum effects in two qubits by means of the $3\times 3$ correlation matrix $R=T^T T$, which is defined by the matrix *T* composed of the two-qubit Stokes parameters $T_{ij}={\textrm{Tr}}[\rho (\sigma _{i}\otimes \sigma _{j})]$, which are the mean values of the Pauli matrices $\sigma _{i}$ ($i=1,2,3$). Superscript *T* denotes transposition. The standard Bloch representation of a general two-qubit state $\rho$ can be given by the elements $T_{ij}$ together with the single-qubit Stokes parameters $u_{i}={\textrm {Tr[}}\rho (\sigma _{i}\otimes I_{2})]$ and $v_{i}={\textrm {Tr[}}\rho (I_{2}\otimes \sigma _{i})]$ as

1 $$ \rho = \frac{1}{4}\Big ( I_{4}+\varvec{u}\cdot \varvec{\sigma }\otimes I_{2}+I_{2}\otimes \varvec{v}\cdot \varvec{\sigma }+\!\!\!\sum \limits _{i,j=1}^{3}T_{ij}\,\sigma _{i}\otimes \sigma _{j}\Big ), $$

where $\varvec{u}=[u_{1},u_{2},u_{3}]$ and $\varvec{v}=[v_{1},v_{2},v_{3}]$ denote the Bloch vectors of the first and second qubits, respectively. Moreover, $\varvec{\sigma }=[\sigma _{1},\sigma _{2},\sigma _{3}]\equiv [X,Y,Z]$, and $I_{n}$ is the *n*-dimensional identity operator.

We analyze in detail a special type of the general states given in Eq. (1). Specifically, we have experimentally generated the polarization Werner states by mixing the singlet Bell state, $| \psi ^{-} \rangle =(| HV \rangle -| VH \rangle )/\sqrt{2}$, with white noise (i.e., the maximally mixed state)^8^:

2 $$ \rho _{\textrm{W}}= p| \psi ^{-} \rangle \langle \psi ^{-} |+\frac{1-p}{4} I_{4},$$

assuming various values of the mixing (noise) parameter $p\in [0,1]$. Here, $| H \rangle$ and $| V \rangle$ denote horizontal and vertical polarization states, respectively. The correlation matrix *R* for the Werner states simplifies to $R(\rho _{\textrm{W}}) = p^2 I_3$.

We also theoretically analyze GWSs, which can be defined by replacing the singlet state $| \psi ^{-} \rangle$ in Eq. (2) by a pure state $| \psi _q \rangle =\sqrt{q}| HV \rangle -\sqrt{1-q}| VH \rangle$ with a superposition parameter $q\in [0,1]$, i.e.,

3 $$ \rho _{\textrm{GW}}(p,q)= p| \psi _q \rangle \langle \psi _q |+\frac{1-p}{4} I_{4}. $$

The state can also be obtained by transmitting a photon in the state $| \psi _q \rangle$ through a depolarizing channel. Note that GWSs is often defined slightly differently, i.e., $\rho '_{\textrm{GW}}(p,q) = p| \phi _q \rangle \langle \phi _q | +(1-p)I_{4}/4,$ where $| \phi _q \rangle =\sqrt{q}| HH \rangle +\sqrt{1-q}| VV \rangle$ instead of $| \psi _q \rangle$ in Eq. (3), as experimentally studied in, e.g., Ref.^9^. A special case of such states, i.e., a modified Werner state, when $| \psi ^{-} \rangle$ is replaced by $| \phi _{q=1/2} \rangle$, is referred to as an isotropic state. Such modifications of the Werner states or the GWSs do not affect their quantum correlation measures.

The correlation matrix *R* for the GWSs, given in Eq. (3), is diagonal and reads

4 $$ R[\rho _{\textrm{GW}}(p,q)] = \begin{pmatrix} 4p^2q(1-q) &{}\quad 0 &{}\quad 0 \\ 0 &{}\quad 4p^2q(1-q) &{}\quad 0 \\ 0 &{}\quad 0 &{}\quad p^2 \end{pmatrix}. $$

We note that the correlation matrices *T* and *R* are in general nondiagonal (including the non-perfect Werner state measured by us experimentally), although they are diagonal for the perfect GWSs states given in Eq. (3). Anyway, as shown in Ref.^59^, an arbitrary state $\rho$ described by a nondiagonal *T*, can be transformed (via a singular-value decomposition) into a state with a diagonal *T* by local unitary operations, thus, without changing its quantum correlations, including those studied below.

## Measures of quantum correlations for Werner and Werner-like states

### Fully entangled fraction and entanglement measures

The FEF^57^ for an arbitrary two-qubit state $\rho$ in Eq. (1) can be defined as^48^:

5 $$ {\textrm{FEF}}(\rho ) = \tfrac{1}{2}\theta ({\textrm{Tr}}\sqrt{R}-1), $$

given in terms the function $\theta (x)=\max (x,0)$. In general, the FEF is only a witness of entanglement; however, for some special classes of two-qubit states, including the GWSs, the FEF becomes a good entanglement measure, and it reduces to the concurrence and negativity:

6 $$ {\textrm{FEF}}[\rho _{\textrm{GW}}(p,q)] = N(\rho _{\textrm{GW}}) = C(\rho _{\textrm{GW}}) = \tfrac{1}{2}\theta \left\{ p [1+4 \sqrt{q(1-q)}]-1\right\} . $$

For completeness, we recall that the concurrence $C(\rho )$ of an arbitrary two-qubit state $\rho$ is defined as^27^: $C(\rho )=\theta (\sqrt{\lambda _1}-\sqrt{\lambda _2}-\sqrt{\lambda _3}-\sqrt{\lambda _4})$, where $\lambda _1 \geqslant \lambda _2 \geqslant \lambda _3 \geqslant \lambda _4$ are the eigenvalues of $\rho (\sigma _2 \otimes \sigma _2)\rho ^*(\sigma _2 \otimes \sigma _2)$, the superscript $*$ denotes complex conjugation, and $\sigma _2$ is the second Pauli matrix. Moreover, we recall the definition of the negativity *N* of a two-qubit state $\rho$, which reads^28^: ${N}({\rho })=\theta (-2\mu _{\min })$ with $\mu _{\min }$ denoting the smallest eigenvalue of $\rho ^{\Gamma }$, i.e., $\min [{\textrm{eig}}(\rho ^{\Gamma })]$, where the superscript $\Gamma$ indicates partial transposition. It is seen that the negativity, concurrence, and FEF reduce to the same function for the GWSs.

Let $p_E(q)$ denote the largest value of the mixing parameter *p* as a function of the superposition parameter *q* for which $\rho _{\textrm{GW}}(p,q)$ is separable. This can be obtained by solving ${\textrm{FEF}}(\rho _{\textrm{GW}})=0$ resulting in:

7 $$ p_E(q)=1/\big [1+4 \sqrt{q(1-q)}\big ],$$

which means that $\rho _{\textrm{GW}}(p,q)$ is entangled iff $p\in (p_E(q),1]$. In the special case of the standard Werner states, Eq. (6) simplifies to

8 $$ {\textrm{FEF}}[\rho _{\textrm{W}}(p)]=N[\rho _{\textrm{W}}(p)]= C[\rho _{\textrm{W}}(p)] = \theta (3p-1)/2, $$

which implies the well known fact^8^ that a given Werner state is separable iff its mixing parameter is $p\in [0,1/3]$.

It should be noted that entanglement measures for general states, given in Eq. (1), depend not only on the correlation matrix *R*, but also on the single-qubit Stokes parameters $\langle \sigma _n^{i}\rangle$ for $n=1,2,3$ and $i=1,2$. It is seen that the FEF is not a universal witness of two-qubit entanglement, because it solely depends on the *R* matrix. Nevertheless, the FEF is a good measure of the entanglement of the GWSs.

### Quantum steering measures

The effect of quantum steering of a two-qubit state refers to the possibility to affect at a distance one qubit (say subsystem *B* of Bob) via local measurements performed on the other qubit (say subsystem *A* of Alice). The quantum steerability of a given two-qubit state $\rho$ can be experimentally tested, assuming that each party is allowed to measure *n* observables in their sites (qubit), by the inequality derived by Cavalcanti, Jones, Wiseman, and Reid (CJWR), which reads^60^:

9 $$ F_n(\rho ,\textbf{r}) =\frac{1}{\sqrt{n}} \left| \sum _{i=1}^n\langle A_i\otimes B_i\rangle \right| \leqslant 1, $$

where $\textbf{r} =\{\hat{r}^A_1,\ldots ,\hat{r}^A_n, \hat{r}^B_1, \ldots , \hat{r}^B_n\}$ is the set of measurement directions with $\hat{r}^A_i,\hat{r}^B_i\in {\mathbb {R}}^3$ (for $i=1,\ldots ,n$) denoting unit and orthonormal vectors, respectively. According to Ref.^61^, the orthogonality of the vectors $\hat{r}^A_i$ is not required, which allows for non-orthogonal measurements to be carried out on the subsystem *A*. Moreover, $A_i = \hat{r}^A_i\cdot \varvec{\sigma }$, $B_i = \hat{r}^B_i\cdot \varvec{\sigma }$, and $\langle A_i\otimes B_i\rangle =\text {Tr}(\rho A_i\otimes B_i)$. A measure of steering can be obtained by maximizing $F_n(\rho ,\textbf{r})$ over the set of measurement directions, i.e., $F_n(\rho )=\max _\textbf{r}F_n(\rho ,\textbf{r})$. More specifically, Costa and Angelo^61^ suggested the following steering measures depending on the number *n* of measurements per qubit:

10 $$ S_n(\rho )={\mathscr { N}}_n \theta [F_n(\rho )-1], $$

where ${\mathscr { N}}_n=[\max _{\rho } F_n(\rho )-1]^{-1}$ is the normalization constant such that $S_n(\rho )\in [0,1]$ for any two-qubit $\rho$. Hereafter, we focus on analyzing the steering measures $S_2$ and $S_3$ (and related quantifiers) in the two- and three- measurement scenarios, denoted as 2MS and 3MS, which correspond respectfully to measuring two and three Pauli operators on qubits of both parties. Costa and Angelo found that these two-qubit steering measures can be compactly written as^61^:

11 $$ S_3 (\rho )= \frac{\theta (c-1)}{\sqrt{3}-1},\quad S_2 (\rho )= \frac{\theta \left( \sqrt{c^2-c^2_{\min }}-1\right) }{\sqrt{2}-1}, $$

respectively, given in terms of $c=\sqrt{c_1^2+c_2^2+c_3^2}$ and $c_{\min }=\min |c_i|$, where $\{c_i\}={\textrm{svd}}(T)$ are singular values of *T*. Note that the original formulas for $S_2$ and $S_3$ in Ref.^61^ were given assuming the diagonal form of the matrix *T*, so $c_i$ were simply given by $T_{ii}$. The steering measures given in (11) can be rewritten in terms of the correlation matrix *R* as follows:

12 $$ S_3(\rho )= \frac{\theta (\sqrt{{\textrm{Tr}}R}-1)}{\sqrt{3}-1},$$

13 $$ S_2(\rho )= {} \frac{\theta \big \{\sqrt{{\textrm{Tr}}R-\min [{\textrm{eig}}(R)]}-1\big \}}{\sqrt{2}-1}. $$

The Costa–Angelo measure $S_3$ of steering in the 3MS is sometimes modified as (see, e.g., Refs.^35,41^):

14 $$S (\rho ) = \sqrt{\tfrac{1}{2}\theta ({\textrm{Tr}}R-1)}, $$

and we also apply this measure in the following, because of a useful property that *S* reduces to the negativity and concurrence for any two-qubit pure states. Note that $S,S_3\in [0,1]$ and they are monotonically related to each other for any two-qubit states:

15 $$ S_3(\rho )=\frac{\sqrt{2S^2(\rho )+1}-1}{\sqrt{3}-1}\le S(\rho ). $$

For the GWSs, described by the correlation matrix *R* given in Eq. (4), we find

16 $$ S[\rho _{\textrm{GW}}(p,q)]= {} \sqrt{\tfrac{1}{2}\theta [8p^2q(1-q)+p^2-1]},$$

17 $$S_3[\rho _{\textrm{GW}}(p,q)]= {} \frac{\theta [p\sqrt{1+8q(1-q)}-1]}{\sqrt{3}-1}. $$

Let $p_S(q)$ denote the largest value of the mixing parameter *p* for a given value of the superposition parameter *q* for which $\rho _{\textrm{GW}}(p,q)$ is unsteerable. Thus, by solving $S(\rho _{\textrm{GW}}) =0$, we have:

18 $$ p_S(q)=[1+8q(1-q)]^{-1/2},$$

which means that a given GWS, $\rho _{\textrm{GW}}(p)$, is steerable assuming three measurements per qubit iff the mixing parameter $p\in (p_S(q),1]$. In the special case of the Werner states, Eq. (16) simplifies to the formulas:

19 $$ S[\rho _{\textrm{W}}(p)] = \sqrt{\tfrac{1}{2}\theta (3p^2-1)},\quad S_3[\rho _{\textrm{W}}(p)] = \frac{\theta (\sqrt{3}p-1)}{\sqrt{3}-1},$$

which imply that $\rho _{\textrm{W}}(p)$ is unsteerable in the 3MS iff $p\in [0,1/\sqrt{3}]$.

Quantum steerability in the 2MS, as based on $S_2$ or related measures, corresponds to Bell nonlocality and it is discussed in detail in the next section.

We note that to quantify steering, assuming three measurements on both Alice’ and Bob’s qubits, we can interchangeably use: $S_3$, defined in Eq. (12), *S*, given in Eq. (14), as well the steerable weight^21^ (as applied in our closely related paper^9^), or the steering robustness^22^ in the 3MS. Indeed, if one of the steering measures vanishes, then all the other measures vanish too. However, the steering measure $S_2$, as defined in Eq. (13) in the 2MS, although it is equivalent to the Bell nonlocality measure *B*, but it is fundamentally different from another steering measure $S_2$ (for clarity denoted here as $S'_2$) studied by us in Ref.^9^, because it corresponds to the case when Alice (Bob) performs two (three) measurements on her (his) qubit. Thus, $S_2(\rho )=0$ (corresponding to vanishing Bell nonlocality of a given state $\rho$) does not imply that also $S'_2(\rho )=0$, which was shown experimentally in Ref.^9^). This is possible because an extra measurement performed by Bob on his qubit, which is allowed in the $S_2'$ scenario, can reveal the steerability of $\rho$.

Finally, it is important to stress that the applied Costa–Angelo measures, because of their invariance under qubit swapping, cannot reflect the directional property of EPR steering that one qubit might be steerable by another, but not vice versa. Specifically, the steering measures $S_2$ and $S_3$ are the functions of some eigenvalues of the correlation matrix $R=T^TT$. By swapping qubits *A* and *B*, one obtains a modified correlation matrix $R'=TT^T$, which, however, has the same eigenvalues as those of *R*. This means that the steering measures are invariant under the qubit-swapping operation, and, thus, describe only two-way-symmetric steering for arbitrary two-qubit states.

However, two-way steering with an asymmetry in the steering strengths^62^ and one-way steering^63^ can be revealed, by some strengthened criteria or measures, including the steering measure $S_{\textrm{LUR}}$ based on local uncertainty relations (LUR), as introduced in Ref.^64^. Note that $S_{\textrm{LUR}}$ cannot be determined from *R*, because its definition requires, in general, the knowledge of not only the correlation matrix *T* (or *R*), but also the vectors $\textbf{u}$ and $\textbf{v}$ for a given density state $\rho$.

The ideal Werner states $\rho _{\textrm{W}}(p)$ and the GWSs $\rho '_{\textrm{GW}}(p,q)$, for any $p,q\in [0,1]$, are unchanged under qubit swapping operation (say $U_{{\textrm{SWAP}}}$). Although, the GWSs $\rho _{\textrm{GW}}(p,q)$, given in Eq. (3), change under qubit swapping, but still can be transformed into a swapping-invariant $\rho '_{\textrm{GW}}(p,q)$ by local unitary operations. Thus, any steering measures are symmetric (including those based on the LUR) for $\rho _{\textrm{W}}(p)$, $\rho '_{\textrm{GW}}(p,q)$, and $\rho _{\textrm{GW}}(p,q)$ with arbitrary *p*, *q*. Of course, this steering-strength symmetry can be slightly broken for experimental states, as we have revealed for the experimental Werner states $\rho ^{\textrm{exp}}_{\textrm{W}}$ reported in Ref.^9^. Note that those states were generated in a setup fundamentally different from that applied in the present paper and reconstructed by a full state tomography. Thus, one can calculate the steering difference $\Delta S_j= | S_{\rm{LUR}}(\rho^{\rm{exp}}_{j,{\rm {W}}})-S_{\rm{LUR}}(U_{\rm{SWAP}} \rho^{\rm{exp}}_{j,{\rm{W}}}U_{\rm{SWAP}})|$ to reveal a potential asymmetry in the LUR-based steering measure from qubit A to B compared to that from qubit B to A, where *j* labels the generated eleven states. Thus, the maximum steering difference for the experimental states of Ref.^9^ can be found to be $\max _j\Delta S_j=0.0016$, which is practically negligible and much less than the corresponding error bars. More importantly, none of those experimental states exhibited one-way steering. As explained above, $S_{\textrm{LUR}}$ cannot be calculated, in general, from *R*, so one cannot calculate $\Delta S_j$ for the experimental data reported here, but one can reasonably assume that $\Delta S_j$ would be negligible as those for the experimental states reported in Ref.^9^.

### Bell nonlocality measures

The Bell nonlocality of a given two-qubit state $\rho$ can be tested by the violation of the Bell inequality in the Clauser–Horne–Shimony–Holt (CHSH) form^65^

20 $$ |\langle {\mathscr {B}}\rangle _{\rho }|\equiv \big |\big \langle \varvec{a}\cdot \varvec{\sigma }\otimes (\varvec{ b}+\varvec{b}^{\prime })\cdot \varvec{\sigma }+\varvec{a}^{\prime }\cdot \varvec{\sigma }\otimes (\varvec{b}-\varvec{b}^{\prime })\cdot \varvec{\sigma }\big \rangle _{\rho }\big |\le 2, $$

where $\varvec{a}, \varvec{a}^{\prime }, \varvec{b}, \varvec{b}^{\prime } \in {\mathbb {R}}^3$ are unit vectors describing measurement settings, and ${\mathscr {B}}$ is referred to as the Bell-CHSH operator. Bell nonlocality can be quantified by the maximum possible violation of the CHSH inequality in Eq. (20) over all measurement settings, which lead Horodecki et al. to the following analytical formula^58^

21 $$ \max _{\nu }\langle {\mathscr {B}}\rangle _{\rho }=2\sqrt{M(\rho )}, $$

where the nonnegative quantity $M(\rho )$ is the sum of the two largest eigenvalues of $R(\rho )$. The CHSH inequality in (20) is satisfied iff $M(\sigma )\le 1$. For a better comparison with other measures of quantum correlations defined in the range [0,1], the Bell nonlocality measure of Horodecki et al.^58^ can be given by (see, e.g.,^41,48,66^)

22 $$ B(\rho ) = \sqrt{\theta [M-1]} =\sqrt{\theta \big \{{\textrm{Tr}}R -\min [{\textrm{eig}}(R)]-1\big \}}, $$

or, equivalently, as^61^

23 $$ B'(\rho ) = \frac{\theta [\sqrt{M}-1]}{\sqrt{2}-1} = \frac{\theta \big (\sqrt{{\textrm{Tr}}R-\min [{\textrm{eig}}(R)]}-1\big )}{\sqrt{2}-1} = S_2(\rho ), $$

which guarantee that $B,B'\in [0,1]$. It is seen that $B'$ is exactly equal to the steering measure $S_2$, given in Eq. (13), in the 2MS.

Hereafter, we apply both nonlocality measures because their specific advantages. In particular, as shown explicitly below, $B'$ depends linearly on the mixing parameter *p* of the Werner states and GWSs, thus its experimental estimation results in smaller error bars compared to those of *B*. On the other hand, *B* is equal to the negativity and concurrence^37,66^, but also to the steering measure *S* and the FEF:

24 $$ B(| \psi \rangle ) = S(| \psi \rangle )={\textrm{FEF}}(| \psi \rangle ) = C(| \psi \rangle )=N(| \psi \rangle )=2|ad-bc|,$$

for an arbitrary two-qubit pure state $| \psi \rangle =a| HH \rangle +b| HV \rangle +c| VH \rangle +d| VV \rangle$, where *a*, *b*, *c*, *d* are the normalized complex amplitudes. This useful property of *B* is not satisfied for $B'$. We also study *B* to enable a more explicit comparison of our present experimental results with those in our former closely related papers^9,48^. Anyway, *B* and $B'$ are monotonically related to each other:

25 $$ B'(\rho )=\frac{\sqrt{B^2(\rho )+1}-1}{\sqrt{2}-1} \le B(\rho ). $$

The Bell nonlocality measures *B* and $B'$ for the Werner states read

26 $$ B[\rho _{{\textrm{W}}}(p)] = \sqrt{\theta (2p^2-1)},\quad B'[\rho _{{\textrm{W}}}(p)] = \frac{\theta (\sqrt{2}p-1)}{\sqrt{2}-1}, $$

which explicitly shows that the states are nonlocal iff $p>1/\sqrt{2}$. By comparing Eq. (26) with Eq. (8), it is clearly seen that the Werner states for the mixing parameter $p\in (1/3,1/\sqrt{2})$ are entangled, although they do not violate the CHSH inequality, as was first predicted in Ref.^8^. For the GWSs, formulas in Eq. (26) generalize to:

27 $$ B[\rho _{{\textrm{GW}}}(p,q)]= \sqrt{\theta \left\{ p^2 [1+4 q(1-q)]-1\right\} }, $$

28 $$ B'[\rho _{{\textrm{GW}}}(p,q)]= \frac{\theta [p\sqrt{1+4 q(1-q)}-1]}{\sqrt{2}-1}, $$

Let $p_B(q)$ denote the largest value of the mixing parameter *p* for a given value the superposition parameter *q* for which $\rho _{\textrm{GW}}(p,q)$ is Bell local. Thus, by solving $B(\rho _{\textrm{GW}})=0$ one finds:

29 $$ p_B(q)=[1+4q(1-q)]^{-1/2}, $$

which means that $\rho _{\textrm{GW}}(p,q)$ is Bell nonlocal if $p\in (p_B(q),1]$. This function reduces for $q=1/2$ to the well-known result that the Werner state violates the CHSH inequality iff the mixing parameter $p\in (1/\sqrt{2},1]$^8^.

**Figure 1:**
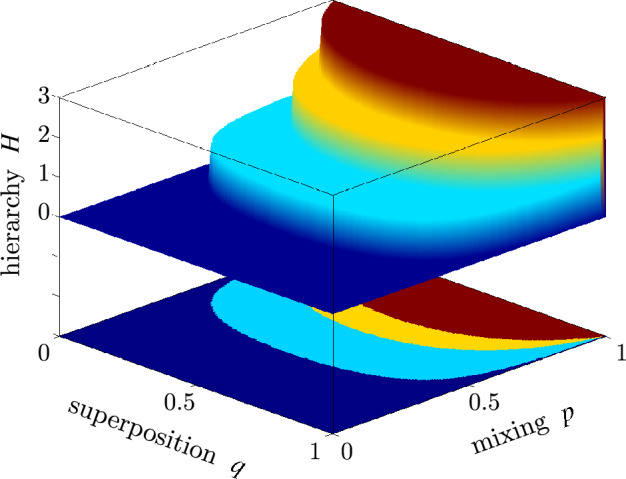
Hierarchy of quantum correlations of the generalized Werner states: The hierarchy parameter $H[\rho _{\textrm{GW}}(p,q)]$, defined in Eq. (33), versus the superposition (*q*) and mixing (*p*) parameters. A given GWS, $\rho _{\textrm{GW}}(p,q)$, is separable if $H(\rho _{\textrm{GW}})=0$, entangled if $H(\rho _{\textrm{GW}})\ge 1$, steerable in the 3MS if $H(\rho _{\textrm{GW}})\ge 2$, and Bell nonlocal (and steerable in the 2MS) if $H(\rho _{\textrm{GW}})=3$.

### Hierarchy of quantum correlations

The following hierarchy of the discussed quantum correlation measures hold for a general two-qubit state $\rho$:

30 $$ B(\rho ) \le S(\rho ) \le {\textrm{FEF}}(\rho ) \le N(\rho ) \le C (\rho ), $$

or, equivalently,

31 $$ S_2(\rho ) \le S_3(\rho ) \le {\textrm{FEF}}(\rho ) \le N(\rho ) \le C (\rho ), $$

We also note that $S_2(\rho ) \le B(\rho )$ and $S_3(\rho ) \le S(\rho )$. The inequalities in (30) for the GWSs reduce to

32 $$ B(\rho _{\textrm{GW}}) \le S(\rho _{\textrm{GW}}) \le {\textrm{FEF}}(\rho _{\textrm{GW}}) = N(\rho _{\textrm{GW}}) = C(\rho _{\textrm{GW}}). $$

To visualize this hierarchy, we define the following hierarchy parameter of quantum correlations for the GWSs,

33 $$ H(\rho _{\textrm{GW}}) = \chi [B(\rho _{\textrm{GW}})] + \chi [S(\rho _{\textrm{GW}})] + \chi [{\textrm{FEF}}(\rho _{\textrm{GW}})] = \chi [S_{2}(\rho _{\textrm{GW}})] + \chi [S_{3}(\rho _{\textrm{GW}})] + \chi [{\textrm{FEF}}(\rho _{\textrm{GW}})], $$

which is given in terms of the Heaviside function $\chi (x)$ equal to 1 for $x>0$ and zero for $x\le 0$. This parameter is plotted in Fig. 1 as a function of the parameters *p* and *q* uniquely specifying $\rho _{\textrm{GW}}(p,q)$.

## Methods

**Figure 2:**
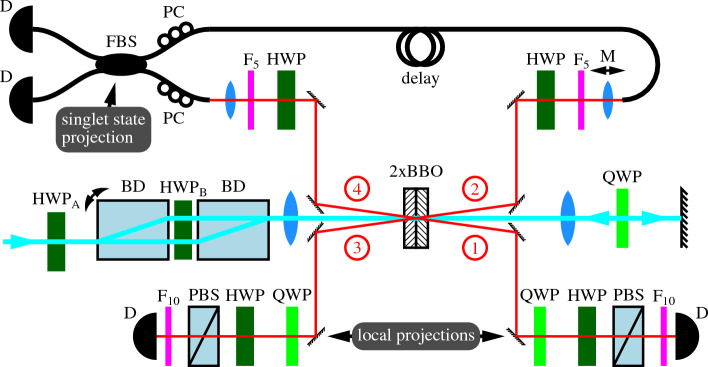
Schematic depiction of the experimental setup. Individual components are labelled as follows: *HWP* half-wave plate, *QWP* quarter-wave plate, *D* detector, *BD* beam displacer, *PBS* polarizing beam splitter, *BBO* $\beta$-barium-borate crystals, *M* motorized translation, *F*$_{5,10}$ 5, 10 nm-wide bandpass filters, *FBS* fiber beam splitter, *PC* polarization controller. Photons generated during the forward (backward) propagation of pump photons through the BBO crystals are labelled as 1 and 2 (3 and 4).

The experiment is implemented on the platform of linear optics with qubits encoded into polarization states of discrete photons. These photons are generated in the process of spontaneous parametric down-conversion occurring in a cascade of two Type-I BBO crystals in the Kwiat et al. configuration^67^. A femtosecond fundamental laser pulse is frequency doubled to 413 nm and pumps the crystal cascade on its way there and back (as depicted in Fig. 2). Each time the pulse impinges on the crystals, a pair of photons can be generated in the polarization singlet Bell state. To achieve a high degree of entanglement, the pumping pulse is diagonally polarized (by the half-wave plate HWP$_{\textrm{A}}$) and subject to a polarization dispersion line^68^. In our case, this dispersion line is implemented by two beam displacers (BDs) enveloping HWP$_{\textrm{B}}$. The photons generated, while the pulse propagates forward are labelled 1 and 2 while the photons generated in the pulses second-time travel through the crystals are denoted 3 and 4.

The investigated state is encoded both into photons 1 and 2 (the first copy) and into photons 3 and 4 (the second copy). A collective measurement on both copies is then performed by projecting photons 2 and 4 onto the singlet Bell state using a fiber beam splitter (FBS) followed by post-selection onto coincidence detection on its output ports. The remaining photons 1 and 3 are projected locally by means of the sets of quarter and half-wave plates (QWPs and HWPs) and polarizing beam splitters (PBSs). We recorded the number of four-fold coincidence detections for various settings of the wave plates; namely, for all combinations of the projections onto the horizontal, vertical, diagonal, anti-diagonal, and both circular polarization states. We have subsequently calculated the expectation values of the Pauli matrices, that is $A_{ij}={\textrm{Tr}}[\rho _1\rho _2\Pi \sigma _i\sigma _j]$, where $\Pi =-4| \psi ^{-} \rangle \langle \psi ^{-} |$. Note that this formula is almost identical to the one in our previous paper^48^, except that in the paper instead of the $\Pi$ projection, the $1-\Pi$ projection was applied there.

When adjusting the setup to generate the requested Bell state (or the maximally mixed state), we have tuned the polarization of the pump beam, so that locally the generated photons have equal probabilities to be horizontally and vertically polarized. (The probability for a single photon being horizontally polarized is $p_H=0.50\pm 0.03$.) Balancing these probabilities for the horizontal and vertical polarizations implies also balancing in any single-photon polarization basis. Note that the single-photon state is fully incoherent, because the other photon from a pair is ignored and, hence, mathematically one traces over its state. As a consequence, we can consider $B_{ij}={\textrm{Tr}}[\rho _1\rho _2 I_{4}\sigma _i\sigma _j] \approx 0$. With respect to that we conclude that the prepared two copies of the Bell state are balanced enough to warrant the replacement of $1-\Pi$ by $\Pi$. This is also supported by the fact that the numerically closest Bell state producing the observed values for the three measures has its parameter $q=0.474$ – see Eq. (38) and comments in the surrounding paragraph.

Despite narrow frequency filtering on all photons (see the parameters of the bandpass filters in Fig. 2) and a relatively thin crystal cascade of twice 1 mm, there is a generation-time jitter, which causes the visibility of two-photon interference on the FBS to decrease. We have performed a calibration measurement that reveals that 56.7% of the photons do not interfere on FBS. Moreover, the laser power fluctuates over time yielding variable rates of photon-pairs generation. In order to compensate for these two effects, we have performed all the measurements in the two regimes with a temporal delay between photons 2 and 4: (a) tuned for interference and (b) detuned (controlled by the motorized translation M). These two measurements together with the calibration measurement allow us to estimate the net probability of the two copies of the investigated state to pass simultaneously the Bell-state projection on the FBS, as well as the local polarization projections resulting in a four-fold detection event. With the repetition rate of the laser pulse of 80 MHz, we achieve about 1 such an event per 5 minutes.

While the crystals generate two copies of the singlet Bell state, we can readily modify the detection electronics to effectively perform the measurement on the two copies of a maximally mixed state. So far the coincidence window, i.e., the time within all photons must be detected to be considered a coincidence event, had to be very narrow (5 ns) to assure detection of photon pairs originating from a single laser pulse. By considerably widening that window by several orders of magnitude, we effectively aggregate also detections that are completely unrelated and mutually random. This way, the observed state becomes effectively white noise.

Having all the measurements performed on a pure entangled state (two copies of the singlet Bell states) as well as on the maximally mixed state (i.e., the two copies of the maximally mixed state), we can easily interpolate the results for any Werner state with mixing parameter *p*. In order to do so, we make use of the fact that when two polarization states of single photons interact on a beam splitter and one of them is being a maximally mixed state, the resulting probability of coincidence detection is independent of the state of the other photon. As a result, we interpolate the measurement for any Werner state by combining with probability $p^2$ the outcomes observed on two copies of maximally entangled states and with probability of $1-p^2$ the results observed on a maximally mixed state.

Note that, contrary to reconstructing the *R* matrix, there is no experimental advantage of reconstructing the $3\times 3$ matrix $T\equiv T_3$ compared to a full QST of a two qubit state $\rho$, which corresponds to reconstructing the $4\times 4$ matrix $T_4=[\langle \sigma _n\otimes \sigma _m\rangle ]$ for $n,m=0,\ldots ,3$, where $\sigma _0=I_2$ is the qubit identity operator. It might look that reconstructing all the 9 elements of $T_3$ is much simpler than reconstructing 16 (or 15) elements of $T_4$. But this is not the case, because the required types of measurements are the same in both reconstructions. Note that the optical reconstruction $T_3$ for a given two-qubit polarization state $\rho$ is usually based on projecting $\rho$ on all the eigenstates of the three Pauli operators for each qubit, i.e., projections onto the six polarization single-qubit states (so 36 two-qubit states): diagonal ($|D\rangle$), antidiagonal ($|A\rangle$), right- ($|R\rangle$) and left-circular ($|L\rangle$), horizontal ($|H\rangle$), and vertical ($|V\rangle$). Analogously, a standard QST of $\rho$ also corresponds to reconstructing $T_4$ via the same 36 projections as those for $T_3$, and the single-qubit identity operator is given by $I_{2}=|H\rangle \langle H|+|V\rangle \langle V|$. So, the required measurements for reconstructing $T_3$ and $T_4$ are the same, but only their numerical reconstructions are different, although can be based on exactly the same measured data.

## Results

**Figure 3:**
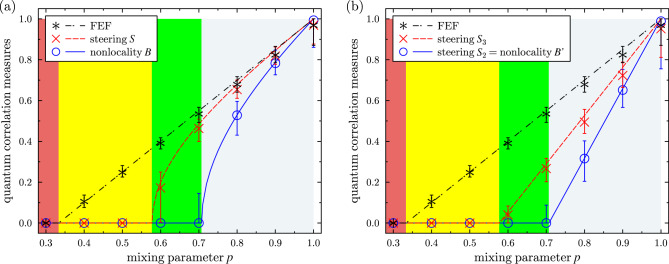
Experimental demonstration of the hierarchy of quantum correlations of the Werner states without full QST: the Bell nonlocality measures (**a**) *B* and (**b**) $B'=S_2$ (solid blue lines and curves), the 3MS steering measures (**a**) *S* and (**b**) $S_3$ (dashed red), and (**a**,**b**) the FEF (dot-dashed black lines) shown versus the mixing parameter *p*. Symbols depict experimental results and curves represent theoretical predictions.

**Table 1:** Quantum correlation measures for the experimental and theoretical Werner states plotted in Fig. 3a, including measures of Bell nonlocality (*B*) and steering (*S*) in the 3MS, and the FEF. Experimental values are listed together with their asymmetric errors in square brackets.

*p*	*B*		*S*		FEF	
	Theory	Experiment	Theory	Experiment	Theory	Experiment
0.3	0.000	0.000	0.000	0.000	0.000	0.000
0.4	0.000	0.000	0.000	0.000	0.100	$0.106 [-{0.030},+{0.031}]$
0.5	0.000	0.000	0.000	0.000	0.250	$0.248 [-{0.022},+{0.034}]$
0.6	0.000	0.000	0.200	$0.172 [-{0.168},+{0.078}]$	0.400	$0.391 [-{0.028},+{0.027}]$
0.7	0.000	$0.000 [-{0.000},+{0.145}]$	0.485	$0.463 [-{0.064},+{0.046}]$	0.550	$0.534 [-{0.041},+{0.032}]$
0.8	0.529	$0.528 [-{0.098},+{0.068}]$	0.678	$0.654 [-{0.043},+{0.047}]$	0.700	$0.679 [-{0.038},+{0.038}]$
0.9	0.787	$0.783 [-{0.057},+{0.064}]$	0.846	$0.818 [-{0.041},+{0.045}]$	0.850	$0.824 [-{0.038},+{0.042}]$
1.0	1.000	$0.993 [-{0.133},+{0.007}]$	1.000	$0.969 [-{0.092},+{0.030}]$	1.000	$0.969 [-{0.098},+{0.031}]$

**Figure 4:**
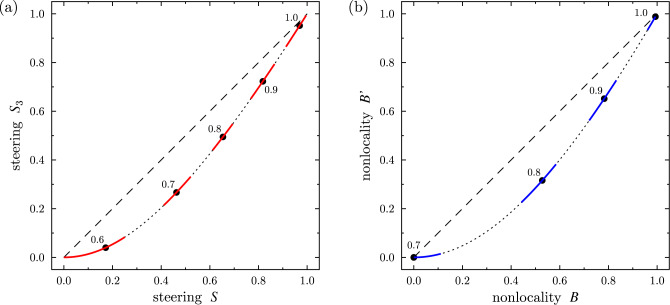
Experimental and theoretical predictions of different measures: (**a**) $S_3$ vs *S* quantifying steering in the 3MS and (**b**) $S_2=B'$ vs *B* describing Bell nonlocality and, equivalently, steering in the 2MS. Symbols depict the measures calculated for the experimental Werner states for the indicated values of the mixing parameter *p*. The error bars are marked by solid red curves that follow the dotted curves. Arbitrary two-qubit states lie on the dotted curves. The dashed diagonal lines are added just to show the curvature of the solid curves more clearly.

**Table 2:** The Costa–Angelo measures $S_3$ and $S_2=B'$ of steering in the 3MS and 2MS, respectively, for the experimental and theoretical Werner states plotted in Fig. 3b.

*p*	$S_2$		$S_3$	
	Theory	Experiment	Theory	Experiment
0.3	0.000	0.000	0.000	0.000
0.4	0.000	0.000	0.000	0.000
0.5	0.000	0.000	0.000	0.000
0.6	0.000	0.000	0.054	$0.040 [-{0.040},+{0.044}]$
0.7	0.000	0.000	0.290	$0.267 [-{0.064},+{0.050}]$
0.8	0.317	$0.316 [-{0.112},+{0.086}]$	0.527	$0.494 [-{0.055},+{0.062}]$
0.9	0.659	$0.652 [-{0.085},+{0.101}]$	0.763	$0.723 [-{0.058},+{0.066}]$
1.0	1.000	$0.989 [-{0.233},+{0.011}]$	1.000	$0.952 [-{0.141},+{0.048}]$

In this section we test the experimental Werner states generated in the setup described in the former section and compare experimental results with theoretical predictions for ideal Werner states. One can calculate the correlation matrix elements $R_{ij}$ following the derivations^48^:

34 $$ R_{ij} = A_{ij} + B_{ij} = {\textrm{Tr}}(\rho _1\rho _2\Pi \sigma _i\sigma _j) + {\textrm{Tr}}(\rho _1\rho _2I_4 \sigma _i\sigma _j), $$

noting that $A_{ij}$ and $B_{ij}$ can be experimentally determined. As a result, the physical correlation matrices $R_{ij}$ of the singlet Bell state and the maximally mixed state were obtained using a maximum likelihood method. First we derive the correlation matrix $R_{|\psi ^-\rangle }$ for the singlet Bell state.

35 $$ R_{|\psi ^-\rangle } = \left( \begin{array}{ccc} 0.971 &{}\quad 0.073 &{}\quad 0.010\\ 0.073 &{}\quad 0.966 &{}\quad -0.009\\ 0.010 &{}\quad -0.009 &{}\quad 0.941\\ \end{array} \right) .$$

Then we evaluated also the correlation matrix for the maximally mixed state corresponding to white noise,

36 $$ R_{I} = \left( \begin{array}{ccc} 0.017 &{}\quad 0.006 &{} \quad -0.007\\ 0.006 &{}\quad 0.013 &{}\quad 0.016\\ -0.007 &{}\quad 0.016 &{}\quad 0.006\\ \end{array} \right) . $$

Using definition (2) we can derive the correlation matrix $R_{\textrm{W}}(p)$ of the Werner states for selected values of the mixing parameter *p* as follows,

37 $$ R_{\textrm{W}}(p) = p^2 R_{|\psi ^-\rangle } + (1-p^2) R_{I}.$$

Now we apply the above-described definitions of the quantifiers of quantum correlations including the defined measures of Bell nonlocality (*B* and $B'=S_{2}$), steering in the 3MS (*S* and $S_3$), and entanglement (FEF) based on this correlation matrix.

Our experimental results are summarized in Tables 1 and 2 and marked by symbols in Figs. 3 and 4. The error bars were derived using a Monte Carlo method following the normal distribution of the correlation matrix components with variance corresponding to the number of detected photocounts. The asymmetry of estimated error bars results from presence of the $\theta$ function in the formulas for the estimated quantities as well as from the requirement on the physicality of the *R* matrices. Figure 3 shows also the theoretically predicted correlation measures plotted with solid curves, which were calculated for the ideal Werner states.

In the theoretical section we considered the Costa–Angelo steering measures $S_2$ and $S_3$ that can be calculated also from the *R* matrix. We evaluated these steering measures using Eqs. (12) and (13). The results are plotted in Fig. 3b. It is clear that these measures linearly depend on the mixing parameter *p*. The nonzero regions of the correlation measures, shown in both panels of Fig. 3, are the same. Experimental results shown in Fig. 3b are also summarized in Table 2.

The original correlation matrices *R* were derived from measured coincidences using two methods of maximum likelihood estimation of Ref.^69^. Both methods lead to the *R* matrices that are essentially the same. Our experimental results shown in Fig. 3 demonstrate a very good agreement with our theoretical predictions. It is clear that $S_2$, $S_3$, and FEF are the most stable measures at least for the Werner states and GWSs by exhibiting the smallest errors because of their linear dependence on the mixing parameter *p*. By contrast to those quantifiers, the measures of steering *S* in the 3MS and of Bell nonlocality (*B*) are much steeper functions and that is why they are much more sensitive to unavoidable fluctuations of measured coincidence counts, as reflected in all the derived quantities. A comparison of the steering measures $S_3$ and *S* and the Bell nonlocality measures $S_2$ and *B* for arbitrary theoretical states and the experimental Werner states are shown in Fig. 4.

In the experiment all imperfections of individual components decrease the resulting correlation measures. Together with the instability and a natural Poisson randomness of the measured coincidences, these effects result in measurement uncertainties. Also our experimentally generated singlet Bell state is not perfect. We tried to simulate all these mentioned imperfections by degrading the input Bell-like state assuming the rest of the measurement to be nearly perfect. These expected imperfections result in a class of generalized states in the form of

38 $$ \rho (p,q) = p |\psi _q^-\rangle \langle \psi _q^-| + (1-p) |\psi _q^+\rangle \langle \psi _q^+|, $$

where $|\psi _q^\pm \rangle = \sqrt{q} |HV\rangle \pm \sqrt{1-q} |VH\rangle$. The correlation measures for our most entangled experimental Bell-like state read: $B = 0.9933$, $S = 0.9691$, and FEF = 0.9685. We found that these results are the most consistent with $\rho (p,q)$ for the parameters $q \approx 0.474$ and $p\approx 0.994$. This implies the purity of this Bell-like state of about 98.9%.

## Conclusions

We reported the detection of quantum correlation measures of two optical polarization qubits without QST. Specifically, we have measured all the elements of the correlation matrix *R* (which is symmetric by definition) for the Werner states with different amount of white noise. These elements correspond to linear combinations of two-qubit Stokes parameters. With the matrix *R*, we were able to determine various measures of quantum entanglement, steerability, and Bell nonlocality of the Werner and Werner-like states.

Most notably, our experiment allows us to show the hierarchy of the tested quantum correlation measures. This means that a given Werner state is separable iff its mixing parameter is $p\le 1/3$. A Werner state for $p \in (1/3, 1/\sqrt{3}]$ is entangled (as revealed by a nonzero FEF), but it is unsteerable and Bell local. Subsequently, a Werner state for $p \in (1/\sqrt{3}, 1/\sqrt{2}]$ is entangled and steerable in the 3MS, but unsteerable in the 2MS, which means that it does not exhibit Bell nonlocality. Finally, a Werner state for $p>1/\sqrt{2}$ is also Bell nonlocal, so steerable even in the 2MS. It is clear that a specific threshold for steerability depends on the number of measurement settings which in our case equal 2 and 3. Different thresholds have been found for different number of measurement settings, see e.g. Refs.^6,9,70^. These regions, separated by the three values of $p = \{1/3, 1/\sqrt{3}, 1/\sqrt{2}\} \approx \{0.333, 0.577, 0.707\}$, are depicted with different background colors in Fig. 3. We have also analyzed theoretically a hierarchy (shown in Fig. 1) of some measures of quantum correlations for generalized Werner states, which are defined as arbitrary superpositions of a two-qubit partially-entangled pure state and white noise.

The problem of detecting measures of quantum correlations is essential to assess their suitability for quantum-information protocols especially for quantum communication and cryptography when considering not only trusted but also untrusted devices. We believe that experimental determination of various measures of entanglement, steering, and Bell nonlocality without full QST, as reported in this work, clearly shows its advantage compared to standard methods based on a complete QST. Specifically, our method relies on measuring only 6 real elements instead of 15 (or even 16) elements in a complete two-qubit QST.

Moreover, experimental studies of a hierarchy of quantum-correlation measures might be useful for, e.g.: (i) testing complementarity relations between various measures, (ii) effective estimations of one measure for a specific value of another measure without full QST, or even (iii) quantifying nonclassicality of single-qubit systems via potentials of quantum correlations.

## Supplementary Information

Supplementary Information.

## Data Availability

All the data necessary to reproduce the results are included in this published article and its digital supplement. We note that all the raw experimental data used in this work were obtained in our experiment reported in Ref.^48^. Of course, their usage and interpretation are very different here compared to the previous work.
